# Effect of TikTok on Self-Harm and Suicidal Behavior in the Adolescent Population: Protocol for a Systematic Review

**DOI:** 10.2196/78600

**Published:** 2025-10-31

**Authors:** Carmen García-Fenollar, Joan Vicent Sánchez-Ortí, Luis Miguel Rojo-Bofill, Rafael Tabarés-Seisdedos

**Affiliations:** 1 Unit of Psychiatry and Psychological Medicine Department of Medicine University of Valencia Valencia Spain; 2 INCLIVA - Health Research Institute Valencia Spain; 3 Center for Biomedical Research in the Mental Health Network National Institute of Health Carlos III Madrid Spain; 4 La Fe Health Research Institute Valencia Spain; 5 University and Polytechnic Hospital La Fe Valencia Spain

**Keywords:** adolescents and young adults, self-harm, social media, suicide, TikTok

## Abstract

**Background:**

Social media use among adolescents and young adults has increased exponentially over the last decade, with TikTok being one of the most popular platforms. In Spain, 61% of adolescents use TikTok, spending an average of 1.5 hours daily on the app. This phenomenon coincides with an alarming increase in the prevalence of self-harm and suicidal behavior among adolescents and young adults. Moreover, suicidal behavior is one of the leading causes of morbidity and mortality in this age group.

**Objective:**

The primary aim of this study is to evaluate the existing evidence on the association of TikTok use with self-harm and suicidal behavior in the adolescent population.

**Methods:**

This systematic review will follow the Preferred Reporting Items for Systematic Reviews and Meta-Analyses (PRISMA) 2020 guidelines to ensure transparency, rigor, and reproducibility. Original studies that evaluate the impact of TikTok use on self-harm and suicidal behavior will be selected. The primary outcome will be the occurrence and prevalence of self-harm and suicidal behavior related to TikTok use among adolescents and young adults. Health science literature databases, including PubMed/MEDLINE, Cochrane, Web of Science, PsycINFO, and Scopus, will be searched. Two researchers will independently select studies that meet the predefined eligibility criteria, and they will extract data from each included study. The risk of bias and methodological quality of the included studies will be assessed using the Risk of Bias in Non-Randomized Studies of Interventions and Joanna Briggs Institute tools, respectively. The methodological characteristics, association measures, and qualitative conclusions of the reviewed studies will be analyzed, and a descriptive synthesis will be presented through tables and graphs.

**Results:**

This systematic review formally began in July 2025, although it has been planned since September 2024. The final systematic review will be performed and reported according to this protocol and the PRISMA guidelines. Initially, the search returned 6126 records, and 3664 records are currently being screened for eligibility. Data from the final included studies will be extracted, collated, and analyzed. The risk of bias and quality of evidence will be determined. A narrative synthesis will be used to summarize the results of the systematic review. When possible, statistical analyses will be presented using graphs and figures. The final systematic review is expected to be published in December 2025.

**Conclusions:**

This systematic review will help to better understand the relationship between TikTok use and self-harm and suicidal behavior among adolescents and young adults. Our findings may support future research, recommendations, and policies in this field, emphasizing the need to incorporate the digital environment as a key factor in adolescent mental health.

**Trial Registration:**

Open Science Framework MX5CJ; https://osf.io/MX5CJ

**International Registered Report Identifier (IRRID):**

DERR1-10.2196/78600

## Introduction

TikTok has established itself as one of the most widely used platforms among teenagers and young adults, characterized by its short video format, a highly personalized recommendation algorithm, and rapid viral dissemination of content [1-4]. These features have been linked to potential mental health risks, including the normalization of self-harming behaviors and the formation of communities around such practices, although specific scientific evidence on TikTok remains limited [5,6].

An increasing number of studies suggest a link between social media use and the deterioration of mental health in adolescents [7,8]. Due to its design and functionality, TikTok may exert a particular influence on the perception and expression of self-harm and suicidal ideation [9]. Documented mechanisms include social contagion, normalization of self-harm, and the creation of online communities that may reinforce vulnerability among adolescents and young adults [10]. Nonetheless, compared with platforms such as Instagram or Facebook, the scientific evidence specifically addressing TikTok remains limited.

Given its highly visual content format and its capacity for rapid viral dissemination, TikTok’s influence on adolescent mental health may differ from that of other platforms. For example, one study found that the use of short-form video platforms was more directly associated with eating disorders among boys and more indirectly associated with these disorders among girls, through body image comparison [11]. Another study highlighted how features such as infinite scrolling and personalized recommendations introduce new forms of reward variability, which may promote compulsive use [12]. Despite these insights, most research to date on social media and mental health in adolescents and young adults, including systematic reviews, has focused on the general impact of social media, such as contagion of behaviors or normalization of behaviors, without analyzing the distinctive characteristics of TikTok. This platform has particular characteristics, such as a highly personalized recommendation algorithm, centering on short audiovisual content, and rapid viral dissemination, that may influence adolescent mental health differently compared to other social media platforms. Consequently, a systematic analysis focused exclusively on TikTok is required. Thus, this study seeks to synthesize the available evidence on the relationship between TikTok and adolescent mental health, thereby providing an updated and specific overview lacking in previous reviews, clearly differentiating what is known about social media in general from what has been documented specifically on TikTok to date.

This context highlights the need to investigate the role of TikTok as a potential risk factor or moderating element in adolescent mental health [13]. This review aims to systematically evaluate the existing literature; the findings could have relevant implications for health care professionals, educators, and policymakers, enabling the design of more effective self-harm prevention and risk-mitigation strategies.

Unlike other platforms, TikTok combines elements of intermittent reinforcement through infinite scrolling and algorithms that prioritize highly personalized content. These mechanisms can intensify repeated exposure to material related to self-harm, increasing the risk of behavioral contagion and hindering users’ emotional regulation. Therefore, it is not just another social network, but an environment with its own dynamics that require a differentiated analysis.

Although adolescence is generally considered to end between the ages of 19 and 21, the psychosocial vulnerabilities linked to social media use and suicidal behavior remain highly relevant during early adulthood, up to the ages of 24 or 25. For example, longitudinal analyses indicate that social media use continues to influence life satisfaction during late adolescence and early adulthood [14]. Furthermore, studies suggest that TikTok plays a significant role in the lives of both adolescents and young adults, affecting areas such as emotional regulation, self-esteem, identity, and mood [6,7,14]. Therefore, in this review, we use the term “adolescents and young adults” to more accurately describe the target population.

In terms of the PECO (population, exposure, comparator, outcome) framework, the objective of this review is to evaluate the current evidence on whether, among adolescents and young adults (population), the use of TikTok (exposure), compared to nonuse or lesser use (comparator), is associated with self-harm and suicidal behaviors (outcome). To achieve this, a review of the available scientific literature will be conducted to better understand the magnitude of this phenomenon, identify patterns of content exposure, and evaluate the possible implications for prevention and intervention strategies.

## Methods

### Study Design

A systematic review of association measures will be conducted. The study protocol was developed following, and the methods and results will be reported in accordance with, the Preferred Reporting Items for Systematic Reviews and Meta-Analyses Protocols (PRISMA-P) guidelines [15-17] (Multimedia Appendix 1). Prior to initiating the project, this protocol was registered on the Open Science Framework platform (MX5CJ). Any amendments or modifications made to the protocol will be described and communicated in the final document.

### Eligibility Criteria

Studies will be selected based on aspects related to study design, population, assessments performed, outcomes, publication type, and language (Textbox 1).

**Eligibility criteria.**

**Inclusion criteria**

- Original studies (randomized controlled trials and quasi-experimental or observational studies)
- Studies that evaluate TikTok use and its association with self-harm and/or suicidal behavior
- Studies that include adolescents and young adults aged 14 to 25 years
- Studies that quantify TikTok use, suicide, and related behaviors using validated assessment instruments

**Exclusion criteria**

- Studies that analyze social media in general (without focusing on TikTok)
- Studies that do not explore the association between TikTok use and self-harm or suicidal behaviors
- Systematic reviews and gray literature

#### Study Type

Any study quantifying the frequency of TikTok use and self-harm and/or suicidal behaviors (ie, randomized controlled trials, quasi-experimental studies, or observational studies) will be included. Studies that do not evaluate the association between TikTok use and self-harm and/or suicidal behavior as a primary objective will be excluded. Studies focusing on social media in general, without specifically evaluating TikTok, will also be excluded. Systematic reviews and gray literature (eg, doctoral theses and book chapters) will not be considered.

#### Population

We will include studies with participants aged up to 25 years, which enables us to cover both adolescents (aged up to 17-19 years) and young adults (aged 18-25 years). This decision was made because both groups are characterized by intensive use of social media and potential exposure to suicide-related risk factors. It is important to note that the protocol specifies that, whenever possible, we will analyze whether the effects of TikTok on suicidal behavior differ between adolescents and young adults. Studies will be included regardless of the sex or ethnicity of the population.

#### Outcome of Interest

Original studies evaluating the association between TikTok use and the onset, maintenance, or exacerbation of self-harm and/or suicidal behavior will be included. Frequency of TikTok use and self-harm/suicidal behavior will be included if they use valid evaluation instruments (ie, self-reported questionnaires or validated scales).

The primary outcomes of this systematic review will be the prevalence of self-harming behavior and suicidal behavior. For the purposes of this review, self-harm refers to intentional self-inflicted injury without suicidal intent, while suicidal behavior refers to self-inflicted injury with the intent to die, regardless of the outcome. Secondary outcomes will include behavioral patterns in searching for and consuming self-harm or suicide-related content on TikTok [13].

#### Publication Status

Only original studies indexed in the consulted databases will be considered, regardless of publication status (eg, preprints, scientific articles, or retracted articles).

#### Language

Only studies published in English will be included, as it is the predominant language in relevant scientific literature.

### Sources of Information

For the information search, various electronic databases of health sciences literature will be considered (from the launch year of TikTok, ie, 2016, to November 2024). Specifically, searches will be conducted on MEDLINE (via PubMed), Cochrane, Web of Science, PsycINFO, and Scopus. Additional data sources will be used, such as the reference lists of studies that meet the inclusion criteria, which will be manually checked to verify whether additional original studies can be included in this systematic review. A search will be conducted using Google Scholar to identify related articles, and the authors of the original studies included will be contacted if necessary.

### Search Strategy

The search strategy will include keywords related to “TikTok,” “self-harm,” “suicide,” and “adolescents,” combined using Boolean operators, such as “(adolescent) AND (TikTok) AND (self-harm).” The review team will develop the first draft of the search and subsequently peer-review it. This strategy will also be adapted for other databases. The draft strategy is provided in Multimedia Appendix 2.

The authors will attempt to replicate the search strategy on TikTok itself and will convert the terms into TikTok hashtags for that purpose. These terms will be combined with hashtags specifically related to self-harm or suicidal behavior. According to the metrics provided by the app, the videos with the most “likes” will be selected for inclusion (eg, trending challenges, content analysis, influencer impact, and hashtag trends). This will be conducted using a TikTok account exclusively dedicated to research in the current year.

A closed list of hashtags related to self-harm and suicidal behavior will be developed based on controlled terminology (Medical Subjects Headings [MeSH] and Emtree), previous literature on digital health, and an exploratory survey on TikTok to identify common synonyms and variations. Searches will be conducted using a TikTok account created exclusively for the research, with no prior history, to minimize algorithmic bias. Each hashtag will be entered individually into the platform’s search bar, recording the date, time, total number of results, and the sorting criteria applied (“most popular” or “most recent”).

Although content with higher interaction (more likes) will initially be prioritized, less frequent hashtags linked in the literature to risky behaviors will also be included to mitigate popularity bias. This broadens the spectrum of exposure, balancing viral content with emerging but clinically relevant content.

The list of hashtags will be created in three stages: (1) reviewing literature and thesauri (MeSH and Emtree); (2) conducting initial exploration on TikTok to find synonyms and variations; and (3) validation by two independent reviewers. All chosen hashtags, along with exclusion criteria, will be recorded in a supplementary file to ensure reproducibility.

For each hashtag, the first 50 videos will be systematically selected according to the established criteria, prioritizing quantitative metrics provided by the platform (views, likes, and comments). Two reviewers will conduct the searches independently and will compare the results, recording screenshots and metadata to ensure the traceability of the process. This procedure will not constitute a systematic literature review, but rather a systematized analysis of social media content, incorporating principles of rigor typical of systematic reviews (predefined criteria, transparent documentation, and bias control) adapted to the dynamic environment of TikTok.

### Study Selection

Once searches have been conducted in all electronic databases, the results will be incorporated into a systematic review management tool. For this purpose, the RefWorks software will be used, and duplicate articles will be removed. Subsequently, two researchers (JVS-O and LMR-B) will independently read the titles and abstracts of all the articles, which will aid traceability, reproducibility, and resolution of discrepancies. In case of discrepancies, conflicting instances will be discussed between the two researchers and submitted to a third researcher (RT-S), who will act as an arbitrator. Articles that do not meet the inclusion criteria, as assessed by both researchers, will be excluded. Both researchers (JVS-O and LMR-B) will read the full articles that were eligible in the previous phase; articles that do not meet the inclusion criteria will be removed and recorded in a list specifying the reasons for their exclusion. Additionally, the references of the selected articles will be reviewed to analyze the possibility of including relevant original articles that were not previously identified.

A flow diagram will be created following the PRISMA guidelines to describe the procedure carried out and number of articles excluded at each step [16].

### Data Collection

After reading the articles, two researchers (JVS-O and LMR-B) will independently collect the data; an experienced researcher (RT-S) will supervise the data collection process. Tables will be designed using Microsoft Word or Microsoft Excel to indicate the main characteristics of the studies. The data extracted from the original studies will include the authors, year, origin, study type, participants, methods, and results.

### Methodological Quality Assessment

The Risk of Bias in Non-Randomized Studies of Interventions (ROBINS-I) tool from Cochrane will be used to assess the risk of bias in nonrandomized studies [18]. The tool asks reviewers to rate the risk of bias in the following domains: confounding, selection of study participants, classification of interventions, deviations from intended interventions, missing data, measurement of outcomes, and selection of reported results. Additionally, the ROBINS-I tool includes an optional judgment on the direction of bias for each domain.

To ensure information validity, methodological quality will be assessed using the Joanna Briggs Institute (JBI) critical appraisal tools [3,19,20], which were selected owing to their detailed and specific approach. Each study will be evaluated using the corresponding scale according to the study type, rating each criterion as “yes” (meets the criterion) or “no” (does not meet the criterion).

### Synthesis of Results

The recommendations of the PRISMA 2020 statement [13] will be followed to present the most relevant results of the search and study selection processes. A narrative synthesis of the main characteristics of the selected original studies will be provided, and all extracted data will be presented in summary tables. Each study will include general, methodological, and transparency elements. A descriptive analysis will be conducted using the frequency and percentage counts of the obtained results, and the results of the methodological quality assessment using the ROBINS-I and JBI tools will be represented in tables or charts (eg, bar diagrams). Finally, the results, strengths, and weaknesses of the original analyzed studies will be discussed.

Given the heterogeneity of the studies included in terms of design, population, variables evaluated, and forms of reporting results, it was not possible to perform a quantitative meta-analysis. Consequently, a narrative/descriptive synthesis was chosen, which allows for rigorous integration of findings and highlights patterns, similarities, and differences between the studies reviewed. This approach is considered the most appropriate for providing a comprehensive overview of the available evidence when quantitative comparison is not feasible.

This review includes studies with participants aged 14 to 25 years, covering adolescents and young adults. This range enables analysis of the life stages of adolescence and the transition to early adulthood. Whenever the data permit, subgroup analyses will be conducted considering age (adolescents aged <18 years versus young adults aged 18-25 years) and gender, aiming to explore differences in exposure patterns and associations with self-harm and suicidal behavior.

### Ethical Considerations

Due to the nature of this systematic review, which is primarily based on the analysis of previous studies, approval from a research ethics committee is not required.

The authors carefully considered the ethical implications of analyzing potentially sensitive content on TikTok in this study. The material examined corresponds exclusively to publicly accessible posts, and no private information was collected, nor was there any interaction with users, so individual informed consent was not necessary. Nevertheless, measures were taken to minimize ethical risks: (1) the inclusion of identifiers that could enable tracing creators was avoided, and (2) self-care guidelines were implemented for the research team in case of exposure to potentially disturbing content. These precautions aim to safeguard both the integrity of indirectly involved users and the well-being of the research team, in accordance with good practices in digital studies.

## Results

This systematic review formally began in July 2025, although it has been planned since September 2024. The final systematic review will be conducted and reported in accordance with this protocol and the PRISMA guidelines [16]. Initially, the search returned 6126 records, and 3664 records are currently being screened for eligibility. Data from the included final studies will be extracted, collated, and analyzed. The risk of bias and the quality of evidence will be ascertained. A narrative synthesis will be used to summarize the findings of the systematic review. Statistical analyses will be presented using graphs and figures to enhance clarity where possible. The final systematic review is expected to be published in December 2025.

## Discussion

### Anticipated Main Findings

This systematic review aims to investigate the proposed link between TikTok use and self-harm or suicidal behavior in adolescents and young adults. Specifically, it endeavors to determine whether exposure to TikTok might affect the onset, persistence, or worsening of these behaviors, while also highlighting gaps and limitations in the current literature [5,10-14].

### Comparison With Previous Work

Previous research has widely linked social media use to impaired adolescent mental health, but evidence focused specifically on TikTok remains limited. Our synthesis aims to clarify whether the structural and functional features of TikTok (eg, short videos, viral trends, and algorithmic recommendations) pose a unique risk compared to other platforms, such as Instagram or Facebook. By situating our findings within this emerging body of evidence, the review will contextualize TikTok’s role in influencing self-harm and suicidal behavior among young people.

### Strengths and Limitations

A key strength of this review is its systematic and transparent approach, guided by PRISMA 2020 and PRISMA-P, which ensure methodological rigor and reproducibility. Furthermore, the use of the ROBINS-I and JBI tools will strengthen the assessment of bias risk and study quality. However, we also anticipate limitations, such as the potential scarcity of high-quality primary studies specifically addressing TikTok, heterogeneity in study designs and outcome measures, the linguistic restriction to English-language publications, and the exclusion of gray literature. These limitations are likely to influence the comprehensiveness of the findings and may also impact both internal validity (risk of publication bias) and external validity (transferability to different sociocultural contexts), which limits the ability to generalize the findings to the entire global adolescent population. Nevertheless, we believe this systematic review will be a valuable synthesis of the available evidence, offering meaningful insights and a solid foundation for future research in the field.

### Future Directions

This review’s findings will lay the groundwork for future research by pinpointing specific knowledge gaps, such as the absence of longitudinal studies, limited focus on protective factors, and superficial examination of TikTok’s content moderation strategies. We also aim to emphasize the importance of interdisciplinary collaboration between clinicians, educators, policymakers, and technology experts to create targeted interventions and preventive measures suited to the digital landscape.

Specifically, we recommend (1) conducting longitudinal studies that explore causality; (2) researching protective factors such as digital resilience and media literacy; and (3) evaluating the effectiveness of content moderation and regulation strategies on TikTok using quasi-experimental designs.

The clinical and public policy implications should be approached with caution, as the available evidence is preliminary and heterogeneous. This review’s results will offer initial hypotheses and guidance but will not establish definitive causal relationships.

### Dissemination Plan

The results of this systematic review will be shared through peer-reviewed scientific publications, presentations at academic conferences, and outreach activities aimed at stakeholders such as health care professionals, schools, and youth organizations. In line with the recommendations of *JMIR Research Protocols*, the communication plan will also include summaries designed for nonspecialist audiences, such as parents and educators, to maximize the potential impact of the findings.

### Conclusion

This systematic review protocol outlines a thorough approach to synthesizing evidence on the link between TikTok use and self-harm or suicidal behavior in adolescents and young adults. The findings are likely to clarify potential risks and support the development of more effective prevention and intervention strategies. Although conclusions will be constrained by the quality and scope of available studies, the outcomes of this review will establish a foundation for future research and policy advice, emphasizing the significance of recognizing the digital environment as a crucial element in adolescent mental health.

## Appendix

Multimedia Appendix 1 PRISMA-P checklist.

Multimedia Appendix 2 Key search terms for various databases.

## Abbreviations

JBI: Joanna Briggs Institute
MeSH: Medical Subjects Headings
PECO: population, exposure, comparator, outcome
PRISMA-P: Preferred Reporting Items for Systematic Reviews and Meta-Analyses Protocols
ROBINS-I: Risk of Bias in Non-Randomized Studies of Interventions
