# Supplementary material for: Effect of TikTok on Self-Harm and Suicidal Behavior in the Adolescent Population: Protocol for a Systematic Review
Source: JMIR Res Protoc. 2025 Oct 31;14:e78600. doi: 10.2196/78600 (PMC12619011; doi:10.2196/78600)
Supplement: Multimedia Appendix 2 [file resprot_v14i1e78600_app2.docx]

**Multimedia Appendix 2**. Key terms for various databases

|  | | **Search** | **Query** |  |
| --- | --- | --- | --- | --- |
| #1 | Population  (child and adolescents) | | “Colleg*”[All Fields] OR “Universit*” [All Fields] OR “Post-secondary” [All Fields] OR “Studen*” [All Fields] OR “Child” [All Fields] OR “adolescent” [All Fields] OR “infant” [All Fields] OR “youth” [All Fields] |  |
| #2 | Social media (TikTok related terms) | | “Social media” [All Fields] OR “Social networking sit*” [All Fields] OR “Online” [All Fields] OR “social network” [All Fields] OR “TikTok” [All Fields] |  |
| #3 | Mental health  (self-harm and suicide) | | “Mental health” [All Fields] OR “Self-harm”[All Fields] OR “Self-harm”[All Fields] OR “self-cut*” [All Fields] OR “self mutilat*”[All Fields] OR “self injur*”[All Fields] OR “suicide”[All Fields] OR “suicidal”[All Fields] OR “suicide attempt” [All Fields] OR “suicide ideation” [All Fields] |  |
| #4 |  | | #1 AND #2 AND #3 | |
